# Therapeutic drug monitoring in patients treated with vancomycin: a single center, prospective, observational, real-world study

**DOI:** 10.1007/s10096-025-05182-w

**Published:** 2025-06-13

**Authors:** Cansu Zeynep Dogan, Emre Kara, Asli Pinar, Kutay Demirkan, Gokhan Metan

**Affiliations:** 1 Department of Clinical Pharmacy, Faculty of Pharmacy, Hacettepe University, Ankara, Turkey; 2 Department of Clinical Pharmacy, Eskişehir City Hospital, Eskişehir, Turkey; 3 Department of Biochemistry, Faculty of Medicine, Hacettepe University, Ankara, Turkey; 4 Department of Infectious Diseases and Clinical Microbiology, Faculty of Medicine, Hacettepe University, Ankara, Turkey

**Keywords:** Vancomycin, Therapeutic drug monitoring, Area under the curve, Nephrotoxicity

## Abstract

**Purpose:**

Therapeutic drug monitoring of vancomycin targeting an AUC/MIC ratio of 400–650 mg*h/L is required to ensure optimal therapeutic efficacy and safety in patients treated with vancomycin. The objectives of this study were to monitor vancomycin plasma peak and trough concentrations to calculate the area under the curve (AUC) to assess target achievement in real-world settings and to evaluate the relationship between AUC and acute kidney injury (AKI).

**Methods:**

In this single-center cohort study, prospectively calculated vancomycin AUC and trough concentrations were extracted from the database and evaluated for achievement of therapeutic ranges for AUC and trough concentrations at a university hospital. Patients were evaluated for the development of AKI according to KDIGO guidelines.

**Results:**

A total of 114 patients were included in the study. Vancomycin loading doses were initiated in 83.3% of patients, and 82.1% of patients received the appropriate weight-based dose. 79.8% of maintenance doses were appropriate. The median (min-max) values for peak, trough, and AUC were 23.25 (3.3-131.8) mg/L, 10.35 (0.6–56.4) mg/L, and 403(49-1786) mg/L*hour, respectively. The majority of values were outside the therapeutic target for both trough (65.3%) and AUC (63.7%). AKI was observed in 15.8% of patients. In patients without AKI, the median (min-max) trough concentration was 9.65 (0.60–45.30) mg/L and the AUC was 370 (49-1390) mg/L*hour.

**Conclusions:**

In this study, only one-third of baseline concentrations were in the therapeutic range and were increased by up to two-thirds with dose adjustments. Therapeutic drug monitoring to achieve target concentrations is critical in patients treated with vancomycin.

## Introduction

Vancomycin is a glycopeptide antibiotic that is active against Gram-positive bacteria, including methicillin-resistant *Staphylococcus aureus* (MRSA), and is used to treat a variety of infections. However, vancomycin treatment requires therapeutic drug monitoring (TDM) to ensure therapeutic efficacy and prevent nephrotoxicity [1, 2, 3].

The first consensus guideline for therapeutic monitoring of vancomycin in adult patients was published in 2009 by a committee representing the American Society for Health-System Pharmacists (ASHP), the Infectious Diseases Society of America (IDSA), and the Society for Infectious Diseases Pharmacists (SIDP). The Committee emphasizes the ratio of area under the curve (AUC) to minimum inhibitory concentration (MIC) over 24 h (AUC/MIC) ≥ 400 mg*hour/L as the primary pharmacokinetic/pharmacodynamic predictor of vancomycin activity. It was recommended that plasma trough concentrations of 15–20 mg/L be used as a marker for optimal vancomycin AUC/MIC in patients with normal renal function and a MIC of ≤ 1 mg/L. Several publications have evaluated the impact of the 2009 guideline recommendations on clinical efficacy and toxicity in patients receiving vancomycin for the treatment of MRSA infections. However, recent data suggest that trough concentration monitoring is associated with an increased risk of nephrotoxicity. Therefore, an AUC/MIC ratio of 400–600 mg*h/L is recommended to achieve clinical efficacy and ensure safety in patients treated for severe MRSA infections [4].

Nephrotoxicity is one of the most common and clinically concerning adverse effects of vancomycin, with its incidence closely associated to drug concentration. Acute kidney injury (AKI) incidence was reported as 21.0% for trough concentrations of 10–15 mg/L, 20.0% for 15–20 mg/L, and 33.0% for ≥ 20 mg/L [3]. Another study investigated the risk of AKI at thresholds of 10, 15, 20, and 25 mg/L and reported that the risk of AKI increased with increasing trough concentrations. A meta-analysis of AKI incidence showed significantly higher incidence rates for trough concentrations ≥ 15 mg/L [5, 6, 7]. These results show a clear relationship between the incidence of AKI and increasing trough concentrations. Recent evidence suggests that vancomycin-induced AKI correlates better with AUC than with trough concentrations [8]. Aljefri et al. identified AUC ≥ 650 mg*h/L as a significant risk factor for AKI development and data derived from their meta-analysis demonstrated that AUC-guided therapeutic drug monitoring significantly reduces AKI incidence compared to trough-concentration-guided approaches [9]. The 2020 guideline for MRSA infections recommends that doses be adjusted to achieve an AUC of 400–600 mg*hour/L [10].

The primary objective of this study was to monitor vancomycin plasma peak and trough concentrations and calculate AUC to assess target attainment in real-world settings. The secondary objective was to evaluate the relationship between AUC and AKI according to the KDIGO 2012 guidelines [11].

## Materials and methods

### Study design

This was a single-center cohort study including patients who were followed up by the Department of Infectious Diseases and Clinical Microbiology at a university hospital between July 2020 and September 2023. The study was approved by the Local Ethics Committee. Prospectively calculated vancomycin AUC and trough concentrations were extracted from the database and evaluated for achievement of therapeutic ranges for trough concentrations and AUC (10–20 mg/L and 400–650 mg*h/L, respectively). Values above these concentrations were considered supratherapeutic and values below these concentrations were considered subtherapeutic.

Patients treated with intermittent intravenous vancomycin and ≥ 18 years of age were included in the study. Patients with other vancomycin regimens (oral or continuous infusion), treatment < 72 h, failure to calculate AUC (patients did not have two consecutive steady-state vancomycin plasma concentrations) (defined as missing or incorrect values), chronic kidney disease or renal replacement therapy, increase in serum creatinine (Scr) > 0.5 mg/dL (or ≥ 50% increase) within 72 h before vancomycin initiation, and AKI during the first 24 h of vancomycin treatment were exclusion criteria.

Demographic and clinical data, diagnosis, and indication for vancomycin were collected from the hospital information system database. Comorbidities were classified according to the International Classification of Diseases (ICD). Vancomycin was routinely used with a loading dose of 20–30 mg/kg and a daily dose of 15–20 mg/kg (in actual body weight) given every 8–12 h in patients with normal renal function. The dose of vancomycin was adjusted according to changes in the patient’s creatinine clearance (CrCl) [12]. In our hospital, only vancomycin trough concentrations were monitored before 2020, but vancomycin peak concentration monitoring was introduced in June 2020 according to new guidelines. Because vancomycin reaches a steady state concentration in the blood within 24–48 h, it is recommended that two samples be collected (30–60 min after the end of the 3rd dose infusion for the peak plasma concentration and 30–60 min before the 4th dose for the trough plasma concentration). However, if it is not possible to collect samples as recommended, samples can be collected 30–60 min after the end of the infusion of any subsequent dose (dose x) for the peak concentration and 30–60 min before the administration of dose 1 after this dose x (dose x + 1) for the trough concentration [13].

At our center, blood samples for vancomycin were routinely collected in EDTA-containing tubes, centrifuged at 4000 rpm for 5 min, and plasma was stored at -20 °C until analysis. Vancomycin concentrations were measured once a week in the central laboratory of our hospital. Plasma vancomycin concentration measurements were performed using a validated method (LC79010, Eureka Lab Division, Ancona, Italy) with liquid chromatography-triple quadrupole mass spectrometry (LC-MS/MS). The LC-MS/MS system consisted of two LC-20AD pumps, a SIL-20AC/XR auto-sampler, a CTO-10ASVP column oven, a DGU-20 A/3R degassing unit, Agilent C18 column, and a triple quadrupole mass spectrometer LCMS-8040 (Shimadzu Kyoto, Japan).

The AUC for vancomycin was calculated prospectively for each patient based on peak and trough concentrations since June 2020 at our center with the “Vancomycin AUC_24_Calculator” in the “Sanford Guide to Antimicrobial Therapy” mobile application. In the calculator, each dose (mg), dose interval (hours), infusion time (hours), measured peak concentration (mg/L), time from infusion start to peak concentration measurement (hours), measured trough concentration (mg/L), time from infusion start to trough concentration measurement (hours) were entered, and the AUC was calculated. All patients received individualized evaluations based on their calculated AUC values during follow-up. For cases requiring dose adjustment, collaborative decisions were made through consultation between infectious disease specialists and primary care physicians, with subsequent recommendations implemented.

According to the KDIGO guidelines, the presence of any of the following is defined as AKI: ≥0.3 mg/dl increase in Scr concentration within 48 h; or ≥ 1.5-fold increase in Scr concentration from baseline, known or estimated to have occurred within the past 7 days; or urine volume less than 0.5 ml/kg/hour over 6 h [11].

### Statistical analysis

Patient demographics, clinical data, and TDM outcomes were analyzed using IBM SPSS Version 23.0 for MacBook. Mean and standard deviation or median and min-max levels were reported for numerical variables and counts and percentages were reported for categorical variables. The normality assumption, one of the parametric test assumptions, was analyzed using the Kolmogorov-Smirnov test and graphical representations. When comparing numerical data, the Student T test was used for normally distributed data, and the Mann-Whitney U test was used for non-normally distributed data. The chi-square test was used to compare categorical data. *p* < 0.05 is considered statistically significant.

## Results

During the study period, 151 patients were treated with vancomycin. Of the initial cohort, 37 patients were excluded (17 for renal impairment, 11 for continuous vancomycin infusion, and 9 for missing/incorrect concentrations), resulting in 114 patients included in the final analysis. The mean (± SD) age of the patients was 55.9 ± 16.2 years. Nearly all patients (96%) had at least one comorbidity, most commonly endocrine, nutritional, metabolic, neoplastic, and circulatory diseases. The primary indications for vancomycin therapy were central nervous system (32.5%) and infective endocarditis (14.0%) (Table 1). The median treatment duration was 13 days (range: 3–207) (Table 1). All patients received at least one additional nephrotoxic medication.

**Table 1:** Demographic and clinical characteristics of the patients

Age (years), mean (SD)	55.9 (16.2)
Gender (male), n (%)	72 (63.2)
BMI (kg/m^2^), mean (SD)	26.5 (4.5)
*Hospitalization Units*	
Medical service, n (%)	54 (47.4)
Surgical service, n (%)	34 (29.8)
Medical intensive care, n (%)	8 (7.0)
Surgical intensive care, n (%)	18 (15.8)
Length of stay in the services (days), median (IQR)	44.5 (45)
*Comorbidities, n (%)*	
Endocrine, nutritional, or metabolic diseases	63 (55.3)
Malignant diseases	53 (46.5)
Cardiovascular diseases	52 (45.6)
Respiratory diseases	24 (21.1)
Central nervous system diseases	17 (14.9)
Musculoskeletal or connective tissue diseases	11 (9.6)
Genitourinary diseases	8 (7.0)
Gastrointestinal disease	6 (5.3)
Mental, behavioral, or neurodevelopmental disorders	6 (5.3)
Blood and blood-forming organs diseases	5 (4.4)
Immune system diseases	5 (4.4)
Eye diseases	5 (4.4)
Ear or mastoid diseases	3 (2.6)
Skin diseases	2 (1.8)
*Indications for vancomycin treatment, n (%)*	
Central nervous system infection	37 (32.5)
Infective endocarditis	16 (14.0)
Bloodstream infection	14 (12.3)
Skin infection	12 (10.5)
Respiratory system infection	11 (9.7)
Musculoskeletal system infection	6 (5.3)
Digestive system infection	4 (3.5)
Blood and blood-forming organs disorder	3 (2.6)
Eye infection	3 (2.6)
Surgical site infection	3 (2.6)
Genitourinary system infection	2 (1.8)
Centeral line associated bloodstream infection	1 (0.9)
Ear infection	1 (0.9)
Pericarditis	1 (0.9)

SD: Standard deviation, BMI: Body mass index, IQR: Interquartile range

Vancomycin treatment was initiated with a loading dose in 95 (83.3%) patients. Of these patients, 78 (82.1%) received the appropriate dose according to weight. The median (min-max) dose for loading was 1875 (900–3000) mg. The median (min-max) total daily maintenance dose was 2318 (900–4800) mg. The number of patients who received the appropriate maintenance dose according to weight was 91 (79.8%). The median (IQR) maintenance dose for those who received a loading dose was 30 (9.5) mg/kg/day, while the median (IQR) maintenance dose for those who did not receive a loading dose was 40 (12.3) mg/kg (*p* < 0.007).

Both peak and trough concentrations for vancomycin were measured a total of 150 times. Vancomycin concentrations were measured twice in 22 patients and three times in 7 patients (Tables 2 and 3). The day of the first concentration was the median (min-max) 5th (2nd-121st) days of treatment. The median (min-max) peak and trough concentrations were 23.25 (3.3-131.8) mg/L and 10.35 (0.6–56.4) mg/L, respectively. The median (min-max) AUC was 403 (49-1786) mg/L*hour. In total, the majority of values were outside the therapeutic target for both trough values (65.3%) and AUC (64.7%) (Table 3).

**Table 2:** First, second, and third concentrations measurement results

Concentration	Median	Minimum	Maximum
First concentrations (*n* = 114)			
Peak, mg/L	23.05	3.3	131.8
Trough, mg/L	10.15	0.6	56.4
AUC, mg/L*hour	392.0	49	1786
Second concentrations (*n* = 29)			
Peak, mg/L	23.9	5.8	88.0
Trough, mg/L	11.0	1.1	31.0
AUC, mg/L*hour	406.0	73.0	1198.0
Third concentrations (*n* = 7)			
Peak, mg/L	24.0	5.0	63.0
Trough, mg/L	13.6	1.6	18.3
AUC, mg/L*hour	431.0	75.0	918.0

**Table 3:** Evaluation of first, second, and third Vancomycin trough concentrations and AUC

	Subtherapeutic range, *n* (%) Trough < 10 mg/L AUC < 400 mg*h/L	In the therapeutic range, *n* (%) Trough 10–20 mg/L AUC 400–650 mg*h/L	Supratherapeutic range, *n* (%) Trough > 20 mg/L AUC > 650 mg*h/L
First concentrations (*n* = 114)			
Trough concentrations	55 (48.2)	36 (31.6)	23 (20.2)
AUC_24_	57 (50.0)	36 (31.6)	21 (18.4)
Second concentrations (*n* = 29)			
Trough concentrations	13 (44.8)	12 (41.4)	4 (13.8)
AUC_24_	14 (48.3)	13 (44.8)	2 (6.9)
Third concentrations (*n* = 7)			
Trough concentrations	3 (42.9)	4 (57.1)	0
AUC_24_	2 (28.6)	4 (57.1)	1 (14.3)
Total (*n* = 150)			
Trough concentrations	71 (47.3)	52 (34.7)	27 (18.0)
AUC_24_	73 (48.7)	53 (35.3)	24 (16.0)

After a total of 29 (19.3%) vancomycin concentration measurements, dose modification was made depending on the AUC (dose increase in 19, dose reduction in 10).

The trough concentration was in the therapeutic range in 26.3% of patients with a loading dose and 57.9% of patients without a loading dose, while it was in the subtherapeutic range in 50.5% of patients with a loading dose and 36.8% of patients without a loading dose (*p* = 0.017). Therapeutic AUC was achieved in 28.4% of patients with a loading dose and 47.4% of patients without a loading dose. AUC was subtherapeutic in 51.6% of patients with a loading dose and 42.1% of patients without a loading dose (*p* = 0.240).

The trough concentration was in the therapeutic range in 33% of patients who received the maintenance dose appropriately and in 26.1% of patients who did not receive the maintenance dose appropriately (*p* = 0.818). The AUC was in the therapeutic range in 34.8% of patients who received appropriate maintenance dose and in 20.0% of patients who received inappropriate maintenance dose (*p* = 0.339).

The median (min-max) trough concentration was 10.3 (0.6–45.3) mg/L in patients with appropriate maintenance dose and 9.6 (2.4–56.4) mg/L in patients with inappropriate maintenance dose (*p* = 0.935). The median (min-max) AUC was 416 (49-1390) mg/L*hour in patients with appropriate maintenance dosing and 332 (114–1786) mg/L*hour in patients with inappropriate maintenance dosing (*p* = 0.563).

The number of patients with vancomycin-related adverse events was 24 (21.1%) (AKI, *n* = 18; thrombocytopenia, *n* = 4; fever, *n* = 3; generalized pruritus/erythema, *n* = 3; neutropenia, *n* = 1; infusion-related phlebitis, *n* = 1). The rate of adverse events was 17.5% (*n* = 10) in patients with subtherapeutic concentrations, 15.4% (*n* = 6) in patients with therapeutic concentrations, and 44.4% (*n* = 18) in patients with supratherapeutic concentrations, respectively (*p* = 0.045). The median (min-max) AUC in patients without adverse events was 374 (49-1786) mg/L*hour, while in patients with adverse events, it was 542 (155–1505) mg/L*hour (*p* = 0.118).

Acute kidney injury during vancomycin treatment was detected in 18 (15.8%) patients. Fourteen (77.8%) out of 18 patients had eGFR = 20–50 mL/min/1.73 m^2^. Hemodialysis was required for 1 (5.6%) patient, and 17 (94.4%) patients had ≥ 1.5-fold increase in serum creatinine. The number of patients who developed renal dysfunction at the end of treatment was 17 (14.9%). At the end of treatment 11 (64.7%) patients had eGFR 20–50 mL/min/1.73 m^2^, 5 (29.4%), patients had eGFR < 20 mL/min/1.73 m^2^ and 15 (88.2) patients had ≥ 1.5-fold increase in serum creatinine.

AKI rate is 12.3% (*n* = 7) in patients with subtherapeutic AUC, 11.1% (*n* = 4) in patients with therapeutic AUC, and 33.3% (*n* = 7) in patients with supratherapeutic AUC, respectively. The median (min-max) trough concentration in patients without AKI was 9.65 (0.60–45.30) mg/L, while in patients with AKI, it was 16.65 (4.40–56.40) mg/L (*p* = 0.014). The median (min-max) AUC in patients without AKI was 370 (49-1390) mg/L*hour, while in patients with AKI, it was 600 (155–1786) mg/L*hour (*p* = 0.019).

The number of patients who were readmitted to the hospital within 30 days was 8 (7.0%) and the 30-day mortality rate was 13.2%.

## Discussion

This is one of the few studies to evaluate trough-peak concentrations, and AUC in patients receiving intermittent vancomycin therapy in a prospective, observational, real-world study. The key findings of this study were that only one-third of initial concentrations were in the therapeutic range and dose adjustments were made up to two-thirds of the time. These rates may be worse in patients treated with routine standard doses (not based on actual body weight) [14]. After initial concentration monitoring, 25.0% of patients required repeat concentration monitoring. Although the primary reason for the need for repeat concentration monitoring is the change in vancomycin dose, other conditions that alter pharmacokinetics (such as changes in body weight and renal function, adverse effects, and presence of critical illness) may also be important reasons [4].

Vancomycin generally reaches a steady state on the 2nd day of treatment, and it is known that the earliest blood concentration can be measured is the 2nd day of treatment [4]. In this study, the median time for the first specimen collection was 5 days. This may be because vancomycin concentration analysis was performed once a week (on Wednesdays) in the laboratory of the study hospital. Physicians send samples 1–2 days before the laboratory working day for sample collection, and these days may fall on the 5-6th day of treatment.

In our study, 31.6% of the trough concentrations and 31.6% of the AUC were within the target range in the first measurement. These percentages were increased in the second and third measurements. The median AUC is within the target range but very close to the lower limit. This finding is surprising considering that most of the patients in this study received a loading dose and loading and maintenance doses were determined by body weight. The observation that most out-of-range measurements were at subtherapeutic concentrations may suggest that vancomycin is preferred at relatively low doses or doses not adjusted according to weight to avoid known adverse effects such as nephrotoxicity [15]. The fact that the dose was increased in two-thirds of the patients who underwent dose adjustment after concentration control further supports this finding. In the study by Bradley et al., approximately one-third of vancomycin orders achieved an AUC/MIC ratio above target, and one-third achieved an AUC/MIC ratio below target [16]. Similarly, a study by Van der Haggen and colleagues found low AUC/MIC ratio target attainment rates with current vancomycin dosing practices. They found that subtherapeutic concentrations were achieved in 68.0% of adults, 76.0% of pediatrics, and 52.0% of neonatal vancomycin orders [17].

In our cohort, patients who received a loading dose were less likely to achieve therapeutic AUC targets compared to those without loading dose (28.4% vs. 47.4%). Subtherapeutic concentrations were more frequent in the loading dose group (51.6% vs. 42.1%, *p* = 0.240). In a retrospective study of 253 patients, the target AUC (400–600 mg*h/L) was reached in 46.0% of patients given a loading dose and 50.0% of patients without a loading dose (*p* = 0.58) [18].

Data from patients with repeated measurements showed that dose adjustments moved the target (for both trough and AUC) closer to the target range. The proportion of concentration results in the therapeutic range also increased with repeated measurements. The reason for this appears to be that clinicians make appropriate dose adjustments according to the concentration of results obtained, and appropriate dose titration can be achieved. This finding supports that therapeutic drug monitoring increases the possibility of individualized treatment according to guideline recommendations. The pharmacodynamic parameter currently considered to be the best predictor of the effective therapeutic activity of vancomycin is the area under the curve over 24 h against the minimum inhibitory concentration (AUC_0 − 24_/MIC) [19]. In our study, the trough concentration and AUC seem to be compatible, but it is difficult to make a clear interpretation because the aim of the study was not to show this correlation.

Nephrotoxicity is a well-recognized adverse effect associated with the use of vancomycin [20]. In our study, as expected, renal problems were the most common adverse reactions associated with vancomycin. The rate of AKI as well as other adverse events associated with vancomycin treatment were significantly higher in patients with supratherapeutic concentrations. The median AUC was also significantly higher in patients with AKI (1.5-fold higher than those without AKI). The incidence of AKI associated with the use of vancomycin ranges from 12.0 to 43.0%, and patients with higher vancomycin exposure are more likely to develop AKI [1]. In a retrospective cohort study by McClure et al., 121 (20.2%) of 600 patients with trough concentrations alone and 87 (15.5%) of 561 patients with AUC/MIC ratio developed AKI during treatment (*p* < 0.05). The supratherapeutic trough concentration was significantly lower in the AUC/MIC group than in the trough concentration only group and the trough concentration remaining in the therapeutic range was significantly higher in the AUC/MIC group [21].

The limitations are that it was a single-center study, and the nephrotoxic drugs administered to the patients were based on medical records. In addition, other risk factors for nephrotoxicity, such as comorbidity and nephrotoxin administration, were not evaluated. The fact that the causative bacteria and treatment response were not evaluated is also an important limitation. Because our hospital does not use Bayesian software, it could not be used to control AUC/MIC estimates.

This is one of the few studies to evaluate trough-peak concentrations, and AUC in patients receiving intermittent vancomycin therapy in a prospective data were collected as part of research infrastructure quality improvement efforts, observational, real-world setting. The study’s key findings revealed that only one-third of initial drug concentrations fell within the therapeutic range. However, through subsequent dose adjustments, we were able to increase therapeutic concentrations by up to two-thirds. These rates may be worse in patients receiving routine standard doses (not based on body weight). These findings highlight the importance of monitoring therapeutic drug concentrations at the start of treatment and adjusting subsequent doses according to the results of this monitoring. It highlights the need for improved vancomycin dosing and monitoring protocols.

## Data Availability

No datasets were generated or analysed during the current study.
